# The Fine LINE: Methylation Drawing the Cancer Landscape

**DOI:** 10.1155/2015/131547

**Published:** 2015-09-13

**Authors:** Isabelle R. Miousse, Igor Koturbash

**Affiliations:** Department of Environmental and Occupational Health, Fay W. Boozman College of Public Health, University of Arkansas for Medical Sciences, 4301 W. Markham Street, Little Rock, AR 72205-7199, USA

## Abstract

LINE-1 (L1) is the most abundant mammalian transposable element that comprises nearly 20% of the genome, and nearly half of the mammalian genome has stemmed from L1-mediated mobilization. Expression and retrotransposition of L1 are suppressed by complex mechanisms, where the key role belongs to DNA methylation. Alterations in L1 methylation may lead to aberrant expression of L1 and have been described in numerous diseases. Accumulating evidence clearly indicates that loss of global DNA methylation observed in cancer development and progression is tightly associated with hypomethylation of L1 elements. Significant progress achieved in the last several years suggests that such parameters as L1 methylation status can be potentially utilized as clinical biomarkers for determination of the disease stage and in predicting the disease-free survival in cancer patients. In this paper, we summarize the current knowledge on L1 methylation, with specific emphasis given to success and challenges on the way of introduction of L1 into clinical practice.

## 1. Introduction

Only about 1% of the genome is comprised of genes while the vast majority is comprised of repetitive elements—retrotransposons, transposons, satellite, and tandem repeats. The first two are also known as mobile or transposable elements, since they are capable of moving within the genome. While the more ancient class—transposons—uses the “cut-and-paste” mechanisms, retrotransposons relocate via an RNA intermediate in a “copy-and-paste” mechanism.

It is becoming increasingly evident that transposable elements are tightly associated with the generation of genetic diversity and can influence the expression of numerous genes. Specifically, transposable elements can affect the integrity of the genome by retrotransposition, resulting in potential insertions and deletions within the coding sequences, as well as genomic rearrangements—by shuffling genomic fragments by 5′ and 3′ transduction and recombination between the homologous elements. Additionally, transposable elements have capacity to affect gene expression by numerous mechanisms, such as providing alternative promoters, silencing by transcriptional or RNA interference (RNAi), and creating cryptic splice sites and polyadenylation signals [1–4].

Two major classes of retrotransposons are long-terminal repeats (LTR) and non-LTR elements. The former are named for their long-terminal repeats flanking the internal proviral sequence on both sides. LTRs are structurally related to exogenous retroviruses, although lacking the ability to move from one cell to another. Two families of these endogenous retroviruses are known to be currently active in mice [5]. Activity of LTR in humans remains controversial, with some reports suggesting that HERV-K is active in the human genome (reviewed in [6]). Non-LTR retrotransposons are presented as autonomous long interspersed nuclear elements (LINE), which include low-copy archaic inactive elements, such as LINE-2 (L2 and LINE-3), and active elements, such as LINE-1 (L1), as well as short interspersed nuclear elements (SINE; Alu—in humans) and SVA [7, 8] that utilize LINE machinery for their mobilization, thus, called nonautonomous.

**Figure 1:**
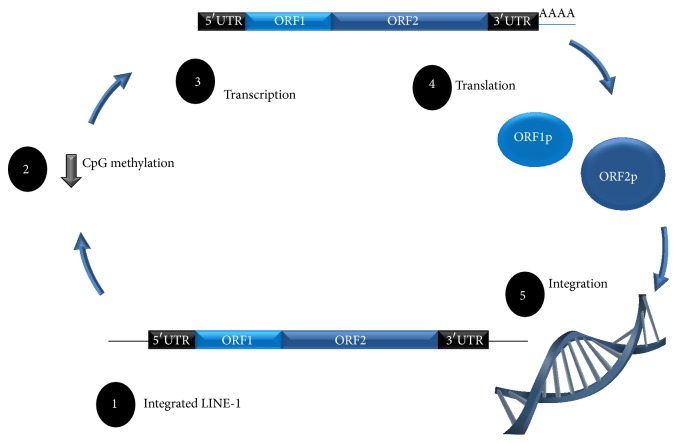
Biology of the LINE-1 element. The LINE-1 element is composed of four units (1). A decrease in silencing methylation marks at CpG dinucleotides (2) may induce an increase in LINE-1 transcription (3). The proteins ORF1p and ORF2p (4) encoded in LINE-1 contribute to its reinsertion in the genome (5).

There are about 516,000 copies of LINE-1 (L1) in the human genome prevalently located within its gene-poor regions, reaching up to 20% of the human genome [9]. However, the vast majority of them are 5′-truncated (0.9 Kb in length on average), contain internal deletions or other mutations, and are thus incapable of retrotransposition. There are, however, ~100 functional full-length L1 in the human genome. They are about 6 Kb in length and contain a 5′-untranslated region (UTR), a bicistronic open reading frame that encodes two proteins—ORF1p and ORF2p—and a 3′-UTR with a poly(A) tail [1] (Figure 1). ORF1p is a 40 kDa protein and is a nucleic acid-binding chaperone. ORF2p is a 150 kDa protein responsible for retrotranspositioning, encoding an endonuclease, reverse transcriptase, and zinc finger-like protein [10, 11]. Earlier studies considered that only ORF2 was needed for successful retrotransposition; however, recent reports clearly demonstrated that both ORF1 and ORF2 are vital for L1 mobilization. The 5′-UTR of L1 contains sense and antisense promoters and binding sites for several transcription factors, including p53, YY1, Runx, SRY, and Socs1 [12–14]. The sense promoter regulates the expression of L1. Although the role of the antisense promoter is still largely unknown, the most recent studies indicate its role in the regulation of transcription of neighboring genes [15] and even those located up to 300 Kb from L1 [16]. Furthermore, the most recent studies show that the L1 antisense promoter is also involved in downregulation of transcription from the L1 sense promoter since the resultant bidirectional transcripts are processed into small interfering RNAs [17], as well as in control over L1 retrotransposition [18].

## 2. Biology of L1

### 2.1. Mechanisms of L1 Mobilization

Propagation of L1 in mammalian genomes occurs via the process of autonomous retrotransposition. Current endogenous retrotransposition activity of some recent L1 elements determines the widespread genomic structural variations within and between populations and variations in normal development, neuronal differentiation, and human cancers [2, 19–22].

Transcription of the L1 full-length mRNA from the internal promoter, mediated by the RNA polymerase II, initiates L1 retrotransposition. This mRNA then is transported to the cytoplasm, where it is translated to ORF1p and ORF2p—L1-encoded proteins that preferentially associate with their encoding RNA [23]. The L1 ribonucleoprotein particle (RNP) is formed then in a* cis-*preference followed by the entrance of RNP into the nucleus [24]. Next, a target-primed reverse transcription (PPRT) occurs. During the TPRT, a single-stranded nick is produced in genomic DNA due to the ORF2p endonuclease activity. This allows for exposing a free 3′-hydroxyl residue that serves as a primer, and a cDNA copy of the associated L1 mRNA is synthesized [1].

Mobilization of L1, thus, occurring via a “copy-paste” mechanism is associated with a number of events, including the development of novel gene promoters, splice sites, polyadenylation signals, dispersing transcription binding splice sites, linking genes in transcriptional networks, and facilitating the evolution of novel traits (reviewed in [3]).

### 2.2. Regulation of L1 (Retrotransposition) Activity

DNA methylation is one of the most important mechanisms for the regulation of genetic information and one of the key mechanisms for silencing repetitive elements (reviewed in [25]). It is a covalent addition of a methyl group to the 5th position of carbon on the cytosine ring in CpG dinucleotides, called CpG sites. It has been estimated that about 56% of these CpG sites are located within repetitive sequences [26]. Taking into account that L1 is the most abundant repetitive element in the genome and that it is heavily methylated in normal somatic cells, one can assume that L1 accounts for the largest portion of methylation.

Silent transcriptional status of L1 has been associated with DNA methylation, specifically within the 5′-UTR, that contains both L1 promoters and is rich in CpG sites. Demethylation of L1 by exogenous stressors, DNA demethylating agents (5-azacytidine), or in disease has been associated with its aberrant transcription [27–29]. DNA methylation, therefore, is considered as a key mechanism for L1 silencing. It has been shown, using embryonic stem cells, that inherited L1 methylation patterns are maintained via utilization of DNA methylation machinery—methyltransferases DNMT1, DNMT3A, and DNMT3B [30].

Other epigenetic mechanisms have also been reported to be involved in the regulation of L1 expression. For example, acetylation and methylation of histones have been implicated in silencing of L1 retrotransposition in embryonic carcinoma cell lines [31]. Accumulating evidence also suggests the role of noncoding RNAs, including Piwi-interacting RNAs (piRNAs), siRNA, and miRNA regulation of L1 [32, 33]. Additionally, a number of other mechanisms, including self-regulation by the L1 antisense promoter, have been proposed and described [18].

## 3. LINE-1 in Cancer

### 3.1. Retrotransposition

When the regulation of normal L1 activity is impaired, retrotransposition events may result in numerous deleterious effects. For instance, it can result in disruption of the ORF of the functional gene, genome amplification, and the development of genomic instability. The first human disease associated with L1 retrotransposition was haemophilia A, stemmed from the independent mutagenic L1 insertions into exon 14 of the* Factor VIII *gene that prevented synthesis of functional coagulation factor [34]. To date, about 100 diseases are known that are associated with L1 retrotransposition, including chronic granulomatous disease, *β*-thalassemia, and diabetes [2, 35, 36].

It has also been hypothesized for a long time that L1 retrotransposition may be associated with cancer development and progression, but the lack of tools needed to detect novel retrotransposition events in human cancers did not allow the support of this hypothesis. The first L1 retrotransposition in cancer was reported by Miki et al. in colorectal cancer and was characterized by insertion of the 3′ portion of L1 into the last exon of the* APC* gene, leading to the disruption of its function [37]. A number of robust and sensitive techniques have been developed since that time to detect retrotransposition events and, up to date, several human cancers have been characterized by the presence of somatic L1 retrotransposition, including colorectal, lung, prostate, and ovarian cancers [38, 39]. However, it still remains largely unknown whether retrotransposition is the driving force of tumorigenesis or merely occurs after tumor initiation. It is certainly without a doubt that a retrotransposition event that occurs within a critical gene, like in the case of* APC* in colorectal cancer [37], can be considered as a driving mechanism. On the other hand, some studies have indicated that L1 insertions may differ in different sections of the same tumor. For instance, the study by Solyom et al. [40] reported that, in about 60% of cases, L1 insertion identified in the first section was not identified in the second section of the same tumor.

### 3.2. Methylation

Loss of global DNA methylation was the first epigenetic alteration demonstrated in human cancers [41, 42]. Subsequent studies have shown that this hypomethylation is not primarily associated with gene-specific methylation, as numerous tumor-suppressor genes in cancers were found in hypermethylated (an often inactivated) status [43, 44]. The following studies clearly demonstrated that global genomic hypomethylation in cancer is associated with the loss of methylation within the TEs, particularly L1 and Alu. Since then, numerous studies were performed demonstrating the loss of L1 methylation in human cancers, and, as of today, L1 hypomethylation has been reported in virtually all human cancers [45].

This hypomethylation can result in a number of unwanted effects associated with aberrant L1 activity and retrotransposition. Also, while L1 is interspersed primarily within gene-poor regions of the genome, its presence within or neighboring the coding sequences can be detrimental. For instance, loss of L1 methylation within its 5′-UTR may result in its aberrant activation and affect the expression of neighboring genes. Hypomethylation of L1 element insertions within the promoters and introns of coding genes may result in aberrant expression of these genes [46]. On the other hand, it has been demonstrated that the presence of repetitive elements facilitates the spreading of methylation into a promoter-CpG island [47]. Altogether, taking into account that about 25% of mammalian promoter regions contain repetitive sequences [48], alterations in DNA methylation within L1 elements may have significant effects on expression of genetic information.

## 4. LINE-1 as a Biomarker

Significant alterations of L1 in human cancers, associated primarily with its increased expression, elevated protein levels, and hypomethylation, together with the very high copy numbers of L1 in the genome, suggest that L1 can be potentially utilized as a diagnostic modality. It is becoming increasingly evident that the methylation status of L1 can be utilized as a prognostic marker in cancer. Indeed, loss of L1 methylation is usually found to be more pronounced in advanced stages of cancer and in metastasis than in the early stages of cancer, or in primary tumors, respectively.

### 4.1. Methylation of L1 as a Prognostic Tool

Growing evidence clearly demonstrates that hypomethylation of L1 is usually associated with poor prognosis and shorter disease-free survival. For instance, a study of 211 lung adenocarcinoma patients concluded that disease-free survival in the group with hypomethylated L1 was significantly shorter than that of the nonhypomethylated group [49]. The results from several studies using cohorts of patients with hepatocellular carcinomas show that hypomethylation of L1 is also inversely correlated with disease-free survival and is associated with poor prognosis [50–52]. Interestingly, the hypomethylated status of L1 was also correlated with higher expression of the* c-MET* oncogene, the gene that contains an L1 insertion within its intron [52].

Importantly, the study by Benard et al. [53] shows that L1 methylation status can serve as an independent clinical prognostic marker in patients with early-stage cancers (stage I-II), as evident from the cohort of patients with rectal cancer.

While, for the vast majority of human cancers, L1 demethylation was associated with poor prognosis, some controversial results exist for melanoma, where L1 hypomethylation was associated with favorable prognosis in stage IIIc patients [54]. However, more recent studies report loss of L1 methylation in regard to poor prognosis and survival in melanoma patients [55, 56].

### 4.2. LINE-1 and Cancer Stage

Accumulating evidence suggests that the methylation status of L1 cannot only be utilized as a prognostic marker but also discriminate between the earlier and later stages of cancer. Extensive research performed in several cohorts of colorectal cancer (CRC) patients indicates that methylation of L1 not only is considerably lower in the tumor compared to adjacent stromal and normal mucosal epithelial cells [57, 58] but notably correlates with the tumor stage in CRC, where the stage 3-4 tumors were characterized by a higher degree of L1 hypomethylation than stage 1-2 tumors [57].

Another study by Park et al. [59], performed on two cohorts of 145 and 179 patients, revealed that decreased levels of L1 can already be identified in human breast samples with atypical ductal hyperplasia/flat epithelial atypia. This suggests that the methylation of L1 can be considered as an early biomarker in cancer diagnosis, as well as clearly providing further evidence of the driving role L1 plays in carcinogenesis. The authors also noted that L1 hypomethylation was associated with negative ER status,* ERBB2(HER2)* amplification, and* p53* overexpression [59]. Similarly, data obtained from the cohort of ovarian cancer patients suggests that L1 hypomethylation is an early molecular event involved in ovarian endometrioid adenocarcinoma and clear cell carcinoma malignant transformation [60].

### 4.3. L1 and the Field for Cancerization

Field for cancerization is the phenomenon characterized by “the occurrence of multifocal and recurrent epithelial tumors that are preceded by and associated with widespread changes of surrounding tissue or organ fields [61].” The role of epigenetic alterations in field or cancerization is well recognized [62–64]. Therefore, hypomethylation of L1, as one of the most frequently observed epigenetic alterations in cancer, may be an important player in the development of field for cancerization. Indeed, the abovementioned study [59] demonstrated that L1 hypomethylation can be detected in breast atypical ductal hyperplasia/flat epithelial atypia. Another study reported correlation between the loss of L1 methylation in normal colon tissue and increased risk for multiple colorectal cancers [65]. Another example of data supporting involvement of L1 in the field for cancerization comes from the study that, among others, evaluated the levels of L1 methylation in normal colorectal mucosa in patients with Lynch syndrome, sporadic colorectal cancer, and familial colorectal cancer [66]. The lowest L1 methylation levels were detected in normal mucosa of patients with familial colorectal cancer, suggesting that L1 methylation status may predispose normal tissue to cancer development [66]. Also, significantly lower levels of L1 were detected in normal mucosa of esophageal squamous cell carcinoma patients with the history of tobacco smoking in comparison with nonsmokers [67].

### 4.4. It Is Better Than the Gene(s)!

It is becoming increasingly evident that the prognostic value of the L1 methylation status might exhibit higher potential than the methylation status of individual tumor-suppressor genes characteristic of a specific cancer. In the recent study, Saito et al. [68] reported that while methylation levels of* APC* and* RASSF1* were significant prognostic factors only in univariate analysis in non-small cell lung cancer, the methylation status of L1 remained significant prognostic factor in multivariate analysis that included age, gender, smoking history, histologic type, and pathologic stage. Moreover, in the same study, L1 methylation also revealed a significant prognostic value for stage IA NSCLC patients in multivariate analysis [68].

Similar findings were reported from the cohort of 217 patients with curatively resected esophageal squamous cell carcinoma, where L1 hypomethylation was significantly associated with shorter survival, while the methylation status of* MGMT* and* MLH1* genes was not associated with patient prognosis [69].

### 4.5. LINE-1 and Metastasis

The role of L1 in tumor's metastatic potential is becoming increasingly recognized [16]. Several recent studies have investigated the L1 methylation status in primary tumors and its distant metastasis. The study by Matsunoki et al. performed in CRC patients did not identify any differences in L1 methylation between the primary tumor and lymph node or distant metastasis [58]. However, the later study by Hur et al. [46], using a larger sample size and more sensitive techniques, reported significantly lower levels of L1 methylation in distant (liver) metastasis, compared to matched primary CRC tissue [46]. Interestingly, they have also shown that the loss of L1 methylation within the intronic region of protooncogenes* MET*,* RAB3IP*, and* CHRMP* results in their reactivation and aberrant expression in CRC metastasis. The recent study by Ikeda et al. [49] reported that vascular invasion in lung adenocarcinoma patients was significantly associated with lower methylation levels of L1.

### 4.6. LINE-1 as a Biomarker in Biological Fluids

Ideal biomarkers should be low-invasive and reflect the pathomorphological changes in the target organs. In this regard, determination of L1 methylation status in biological fluids, such as blood and saliva, is of particular interest. Up to date, a number of studies have attempted to determine the association between the methylation of L1 in leukocytes, peripheral blood mononuclear cells, and buccal DNA and the risk for certain cancers [70–73]. Despite the significant progress achieved in this field in the last year, the results of these studies do not provide a clear picture of L1 methylation status in biological fluids and its association with certain human cancers. For instance, while L1 hypomethylation was detected in peripheral blood leukocytes in patients with gastric cancer [72], L1 hypermethylation in white blood cells DNA was significantly associated with pancreatic cancer [73]. No significant associations in L1 methylation in peripheral blood between melanoma patients and a healthy cohort were found [71]. The most recent meta-analysis performed on 2554 samples from cancer patients and 3553 control specimens identified that although there was a significant association between lower L1 methylation and tumor versus normal DNA, no association for L1 methylation levels in the blood of control and cancer patients was found [74].

### 4.7. LINE-1 as a Biomarker and Prognostic Tool: Expression

While normal adult human tissues usually do not express L1 (or express at very low levels), considerable levels of L1 RNA and protein are found in cancerous tissues [75]. Importantly, several studies report that the extent of L1 expression or protein levels is inversely correlated with the prognosis in pancreatic ductal carcinoma [76] and high-grade breast carcinomas [77], respectively. Another study reports that nuclear expression of both ORF1p and ORF2p is associated with lymph node metastasis in breast cancer and poor patient survival [78].

## 5. Challenges

One of the major challenges is the high degree of variability in L1 methylation between the evaluated samples [57]. Interestingly, the authors extended these findings to include a number of established colon cancer cell lines and have shown that these cancer cell lines also exhibited a large variation in demethylation. This variability was also reported in other studies [79, 80].

Another challenge is variability and certain discrepancies between the studies. For instance, while some studies report L1 hypomethylation in leukemia patients [81], others report no such changes in L1 methylation [82]. Similarly, while one study indicates unfavorable prognosis in melanoma patients associated with L1 hypomethylation [55], another study reports favorable prognosis associated with L1 hypomethylation [54]. These differences can be attributable to a number of factors, including the heterogeneity in human populations involved in these studies; the high degree of tumor heterogeneity, where striking differences in expression and methylation of L1 can be detected in different samples obtained from the same tumor; different assays utilized for the analysis—from COBRA to pyrosequencing and array-based analysis; different L1 regions assayed—5′-UTR, ORF1, and ORF2; and differences in the number of CpG sites analyzed in a given assay. Studies also indicate the possibility of evolutionary age of L1 families influence on the degree of L1 demethylation, where the youngest L1 elements undergo the most dramatic loss of methylation [83, 84]. Also, the most recent report indicates significant differences in L1 methylation from samples collected from the left or right side of the bowel [85], adding an extra level of complexity.

## 6. Concluding Remarks

In the last two decades, L1, the most abundant repetitive element in the human genome, experienced a dramatic switch from being “junk DNA” to being “an important player in the mammalian genomes [86].” Its involvement in numerous important biological processes, and in both health and disease, makes it one of the most interesting subjects. Significant progress is achieved in our understanding of L1 biology and the effects this retrotransposon can exert. L1's role in cancer development and progression is becoming increasingly recognized, given its contribution to global genomic alterations in DNA methylation, expression of genetic information, and retrotransposition. Accumulating evidence suggests that such parameters as L1 methylation status can be potentially utilized as clinical biomarkers for determination of the disease stage and predicting the disease-free survival in cancer patients. However, certain challenges need to be overcome before the introduction of L1 into clinical practice. Additionally, while genetic alterations, such as mutations, are usually irreversible, epigenetic alterations, such as DNA methylation, are potentially reversible and, thus, can provide possible molecular targets for successful cancer therapy.
